# Molecular mechanism of vitiligo treatment by bailing tablet based on network pharmacology and molecular docking

**DOI:** 10.1097/MD.0000000000029661

**Published:** 2022-06-30

**Authors:** Jinming Li, Meng Yang, Yeqiang Song

**Affiliations:** a Shandong University of Traditional Chinese Medicine, Jinan, China; b Department of Cosmetic Dermatology, The Affiliated Hospital of Shandong University of Traditional Chinese Medicine, Jinan, China.

**Keywords:** bailing tablet, molecular docking, network pharmacology, vitiligo

## Abstract

Bailing tablet, a patent Chinese medicine, is widely used to treat vitiligo in China. However, the underlying mechanism of this combined drug in treating vitiligo still remains unclear.

**Objective:**

This study aimed to investigate the pharmacological mechanism of bailing tablet in the prevention and treatment of vitiligo using network pharmacology and molecular docking.

**Methods:**

Genetic data of vitiligo and normal people were obtained by gene expression omnibus (GEO) DataSets database and GEO difference analysis was conducted to obtain differential genes. The main active compounds and corresponding target genes of bailing tablet were collected from Traditional Chinese Medicine Systems Pharmacology Database and Analysis Platform. Combined with the results of GEO difference analysis, the main compounds and corresponding target genes of bailing tablet in the treatment of vitiligo were screened. The network diagram of “traditional Chinese medicine compound target” was constructed by Cytoscape software. According to the differential genes, the core targets with potential therapeutic effect were searched, the protein–protein interaction network was constructed, and the key proteins were explored by topological analysis (CytoNCA). Meanwhile, the core targets were analyzed by biological process (gene ontology) and signal pathway (Kyoto encyclopedia of genes and genomes) function enrichment. Molecular docking technology was adopted to verify the combination of main components and core targets.

**Results:**

A total of 170 active compounds and 1777 prediction targets were screened from 11 traditional Chinese medicines of bailing tablet, of which 65 active components and 68 related prediction targets were closely related to vitiligo. A total of 320 key proteins were obtained by analyzing the topological characteristics of the protein–protein interaction network, mainly including neurotrophic receptor tyrosine kinase 1, tumor protein P53, cullin 3, estrogen receptor 1, etc. The main biological processes involve oxidative stress response, cell response to reactive oxygen species, and reactive oxygen species metabolism. Bailing tablet treats vitiligo mainly by regulating immune inflammation, apoptosis, and autophagy, which involves phosphatidylinositol-4,5-bisphosphate 3-kinase Akt signal pathway, mitogen-activated protein kinase signal pathway, Janus kinase signal transducer and activator of transcription signal pathway, melanin production, and helper T cell (Th)1, Th2, and Th17 differentiation pathway, etc. Molecular docking results showed that the main components could bind to the target protein well.

**Conclusions:**

Based on network pharmacology and molecular docking, the mechanism of bailing tablet in the treatment of vitiligo through multicomponent, multitarget, and multichannel was deeply explored.

## 1. Introduction

Vitiligo is a disease of acquired pigment disorder of skin and/or mucous membranes, with epidermal melanocytes missing, accompanied with skin and leukoplakia. In children and adults, the incidence rate is 0.5% to 2%.^[1]^ Melanocyte death is one of the characteristics of vitiligo. Also, the pathogenesis and etiology of vitiligo are diverse, which has not been confirmed by medical research so far. Most people hold the view that the pathogenesis of vitiligo includes oxidative stress theory, autoimmune hypothesis, neural theory, inherent theory, melanocyte hypothesis of bleeding, etc.^[2]^ The commonly used first-line treatment methods clinically mainly include glucocorticoid, synthetic α-melanocyte-stimulating hormone, calcineurin inhibitor, vitamin D3, phototherapy, surgery, traditional Chinese medicine (TCM), etc. The short-term efficacy of Western medicine in the treatment of this disease is acceptable, while there are certain side effects in the long-term treatment, such as local skin irritation, atrophy, telangiectasia, hormone-dependent dermatitis, and so on. TCM is commonly used for its small side effects, high compliance, and stable curative effects, and its mechanism has been confirmed by modern medicine gradually.

Bailing tablet belongs to a kind of patent Chinese medicine, with the functions of “activating blood circulation and removing blood stasis” and “nourishing blood and dispelling wind.” The main components of the medicine are as follows: *Astragalus membranaceus*, *Angelica dahurica*, *Panax notoginseng*, *Angelica sinensis*, peach kernel, peony bark, safflower, purslane, red peony, Fang feng, and *Atractylodes rhizome*. *Angelica sinensis*, *P notoginseng*, and *A membranaceus* could replenish Qi and blood; peony bark, red peony, peach kernel, and safflower could “activate blood circulation and remove blood stasis”; Fang feng, *Atractylodes macrocephala*, and *A dahurica* could “dispel wind and activate blood circulation”; purslane has the function of “clearing heat and cooling blood.” The combination of the above drugs has the effect of “activating blood circulation and tonifying blood,” and “removing blood stasis and dispelling wind.”^[3]^ Bailing tablet is recommended for the treatment of vitiligo in the consensus of experts in TCM diagnosis and treatment, and its clinical efficacy has been confirmed by many studies.^[4]^ A meta-analysis of 17 clinical studies, including 2832 patients, reported that conventional treatment combined with bailing tablet significantly improved the total effective rate compared with conventional treatment alone, while there are no obvious adverse reactions, confirming the efficacy and safety of bailing tablet.^[5]^ According to modern medicine, bailing tablet can increase photosensitivity, improve the activity of tyrosinase, and improve immunity. It is reported that bailing tablet can improve local skin microcirculation and promote the production of melanocytes to reduce leukoplakia from the periphery to the center.^[6]^ However, the mechanism of bailing tablet in the treatment of vitiligo is not clear. Network pharmacology can systemically reveal the internal mechanism of pharmacology from multiple levels and angles through systems biology, bioinformatics, network science, and other disciplines to have a better guiding direction for clinical diagnosis and treatment.^[7]^

## 2. Materials and methods

### 2.1. Collection of different genes of vitiligo disease

Gene expression omnibus DataSets in the National Center for Biotechnology Information database (http://www.ncbi.nlm.nih.gov) was used to retrieve the word “vitiligo.” The data, with a sample size of >20, were selected to download and the gene data of vitiligo and normal people were obtained. According to the gene expression of samples, differential gene analysis was carried out to screen the upregulated and downregulated genes in patients with vitiligo and healthy people. R software was used for analysis, and the differential genes were obtained based on the absolute value of logFC >1 and the corrected *P* value <.05. The top 20 upregulated and down regulated differential genes were selected to make volcano map and heat map.

### 2.2. Collection and screening of active components of TCM in bailing tablet

Tcmsp (http://tcmspw.com/tcmsp.php/),^[8]^ a database and analysis platform of Traditional Chinese Medicine System Pharmacology (TCMSP), is a platform for running the relationship among drugs, targets, and diseases. TCMSP was used to search and collect the chemical components of 11 herbs (*A membranaceus*, *A dahurica*, *P notoginseng*, *A sinensis*, peach kernel, peony bark, safflower, purslane, red peony, Fang feng, and *Atractylodes lancea*). At the same time, 2 pharmacozoological parameters of oral bioavailability ≥30% and drug (drug-likeliss) ≥0.18 were taken as the criteria. The active components with the main biological effects in bailing tablet were screened.

### 2.3. Screening and prediction of core targets of bailing tablet in the treatment of vitiligo

TCMSP was used to search the DrugBank database to screen the target genes corresponding to TCM compounds. The intersection of drug target genes and disease difference genes was used to predict the core target of TCM compounds in the treatment of vitiligo. TCM, active components of TCM, and core targets were input into Cytoscape (version 3.7.1, http://www.cytoscape.org/) software to construct the network diagram of “bailing tablet–active components–vitiligo disease targets.”

### 2.4. Construction and topology analysis of protein–protein interaction network

Protein–protein interaction network (PPI network) is a database for searching known proteins and predicting PPI that includes direct physical interaction and indirect functional correlation. At the same time, we used Cytoscape to construct PPI network. Gene information was imported into Cytoscape, and HPRD, BIND, DTP, MINT, INTACT, and BIOGRID databases were retrieved through BisoGenet to obtain protein information and select internal nodes and adjacent nodes to make PPI network. CytoNCA in Cytoscape software was used for topology analysis, and the degree centrality (DC) and betweenness centrality (BC) were taken as screening criteria. The minimum value of DC was 61, the minimum value of BC was 600, and the related proteins were obtained.

### 2.5. Gene ontology and Kyoto encyclopedia of genes and genomes enrichment analysis

Gene ontology (GO) analysis is usually divided into 3 categories: biological process (BP), cell component, and molecular function (MF). Enrichment analysis database (David) was used for the 3 types of analysis. *P* value cutoff of .05 and Q value cutoff of 0.05 were taken as the standard. The R software was used for GO enrichment analysis and Kyoto encyclopedia of genes and genomes (KEGG) pathway analysis, and the first 20 enrichment results were selected to draw a bubble diagram. The top 20 KEGG results were taken as the included pathways, the target genes corresponding to each pathway were analyzed, a network diagram was made, the pathways and occurrence times of genes were explained, and the main pathways that play an important role were predicted.

### 2.6. Composition-target molecular docking

Molecular docking technology can simulate and predict the binding mode and affinity between target and drug molecules by computer.^[9]^ Protein structure was collected through protein data bank database (https://www.rcsb.org/), and the 3-dimensional structure of the main components was obtained from the PubChem database (https://pubchem.ncbi.nlm.nih.gov/). The above 2 data were imported into Schrodinger software for pretreatment of target proteins and compounds, then protein ligands were selected to generate lattice files, and finally, the Ligand Docking program was operated to obtain the docking score and the result diagram of molecular docking.

## 3. Results

### 3.1. Differential genes of vitiligo

**Figure 1.**
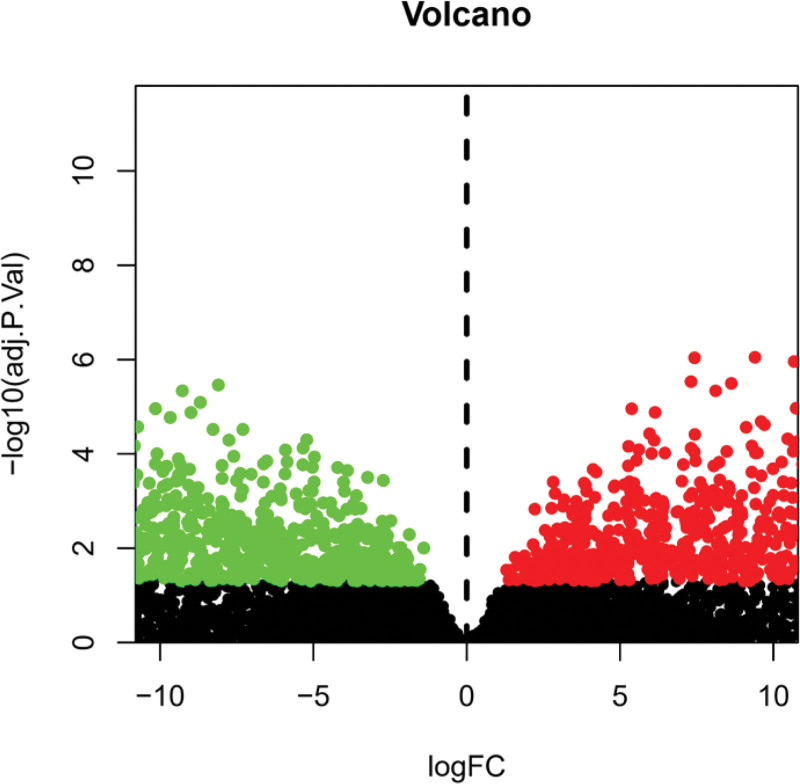
Comparison of different genes in vitiligo. Each point represents a gene, green represents the upregulated genes in the normal group, red represents the upregulated genes in the experimental group, and black represents the genes with no difference between the 2 groups.

**Figure 2.**
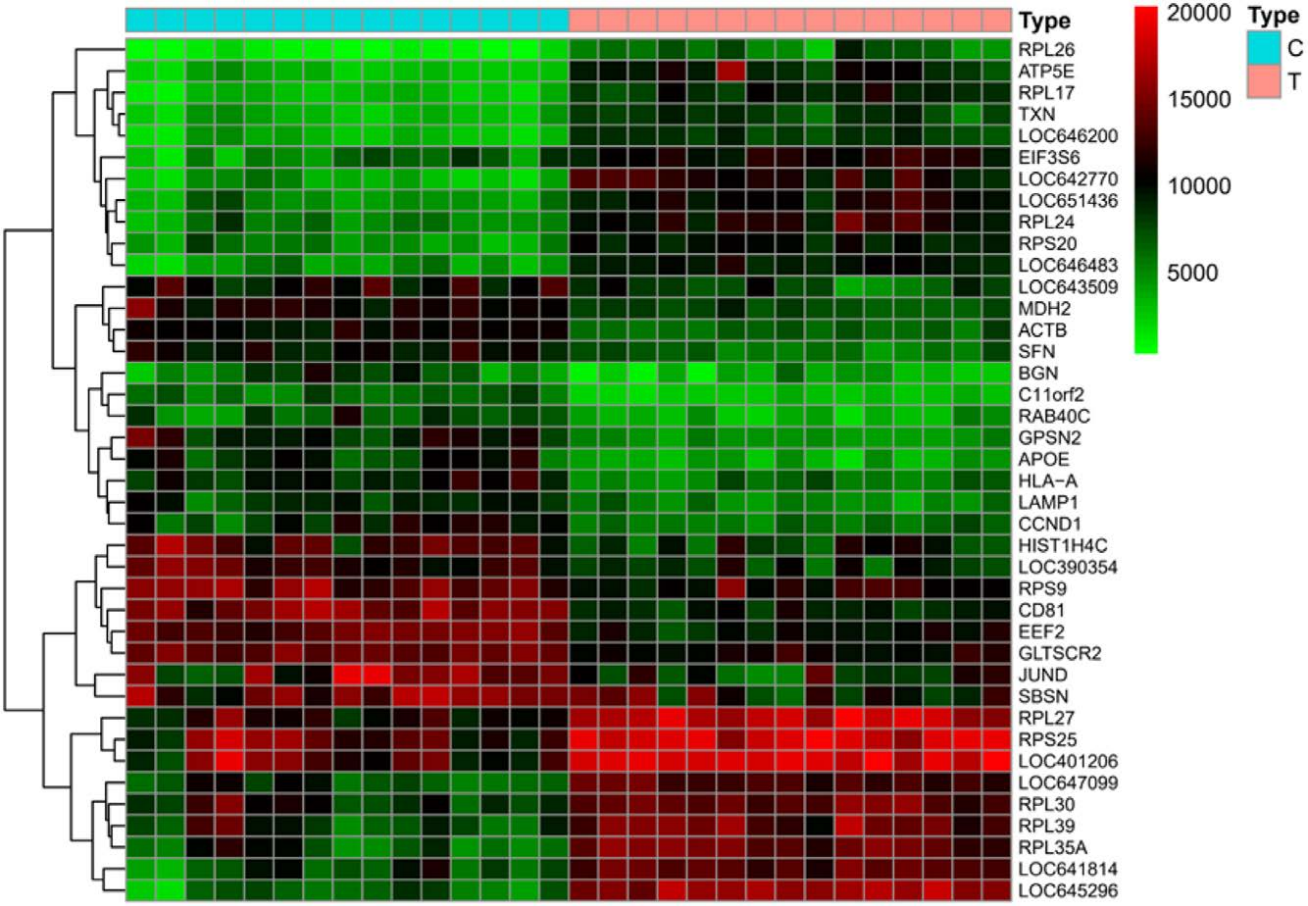
Gene expression in control group (c) and experimental group (T). On the right is the gene name, and the color from low to high represents the gene expression intensity. The top 20 genes with the most obvious upregulation and downregulation are described in this figure.

A total of 30 samples were included, with 15 diseased skin and 15 nondiseased skin. By comparing the related genes of vitiligo in the control group and the experimental group, 7357 differential genes were found, of which 3390 were upregulated and 3967 were downregulated. It is predicted that the pathogenesis of vitiligo is closely related to the expression of these genes (Fig. 1). According to the degree of gene difference, 20 genes with the most obvious upregulation and downregulation were selected to draw the heat map (Fig. 2). Among them, rpl26, atp5e, rpl17, TxN, and loc646200 were highly expressed in the experimental group, while BGN, c11orf2, rab40c, gpsn2, and apoE were poorly expressed in the experimental group.

### 3.2. Screening of active components and targets of TCM

Through TCMSP database, 170 eligible active components and 1777 predicted targets were screened and collected from bailing tablet. After the intersection of the corresponding target of bailing tablet and the disease difference gene, a total of 68 related prediction targets were obtained, including PTGS2, AR, RXR, CXCL10, etc. It was predicted that these may be the main targets of bailing tablet.

### 3.3. Network analysis on “bailing tablet–active ingredient–vitiligo disease target”

**Figure 3.**
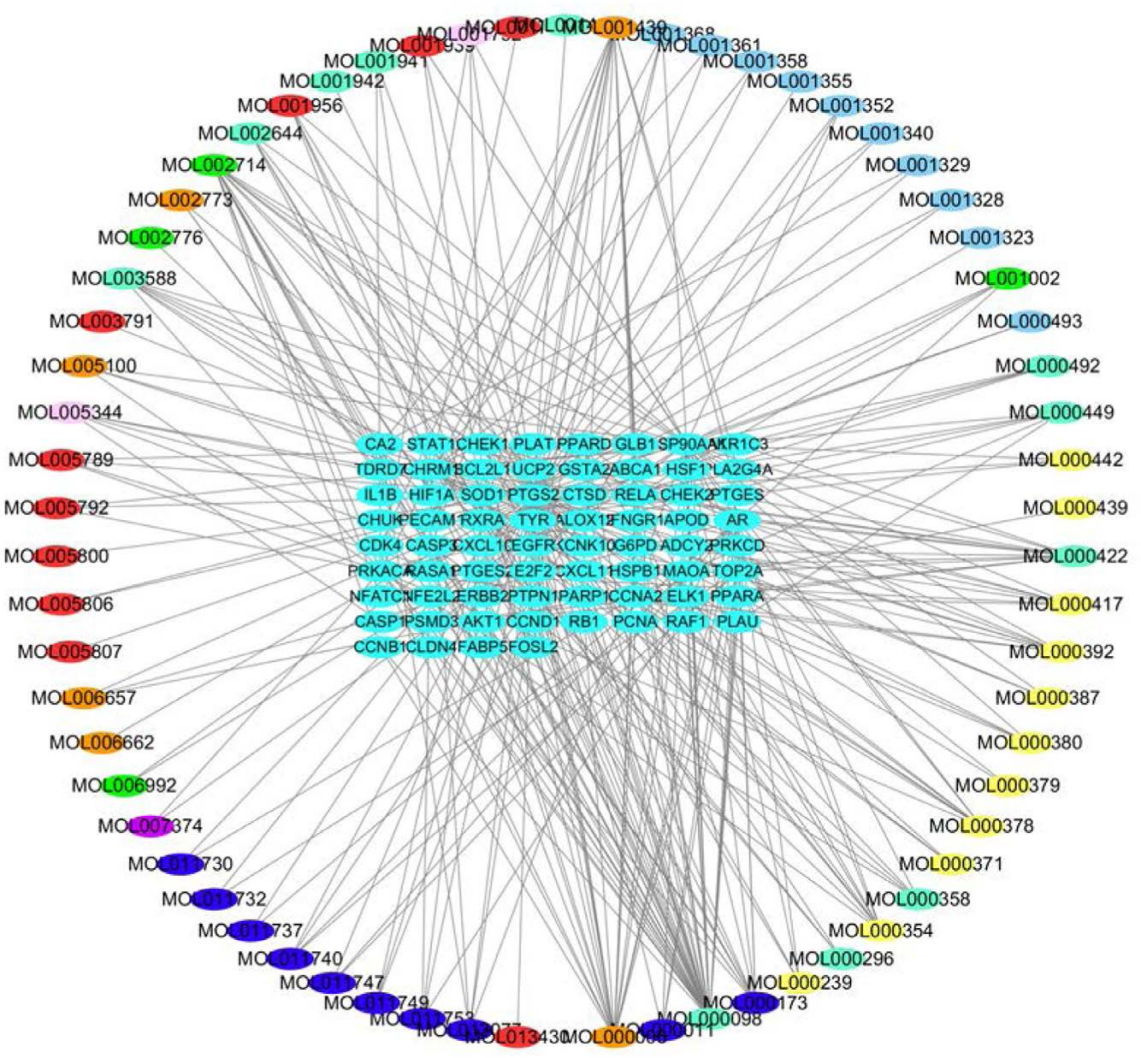
Network diagram of “bailing tablet–active ingredient–vitiligo disease target.” Outer circle: source of active ingredients (yellow: Huangqi; red: Baizhi; Lavender: Sanqi; rose red: Taoren; purple: Mudanpi; orange: Machixian; green: Chishao; dark blue: Fang feng; gray: multidrug); inner rectangle: intersection gene of drug target gene and disease-related gene.

The relationship between 65 active components of bailing tablet and 68 vitiligo treatment targets was visualized by Cytoscape software. A compound can correspond to multiple genes, and a gene can also correspond to multiple compounds. According to the number of active ingredient nodes, the top 10 compounds are quercetin, arachidonic acid, luteolin, baicalein, kaempferol, calycosin, formononetin, prangenidin, phellopterin, and 7-*o*-methyl isomucronulatol (Fig. 3; Table 1).

**Table 1:** Top 10 compounds and the nodes of them.

Component	Mold	Source of TCM	Nodes
Quercetin	MOL000098	Honghua, Huangqi, Machixian, Mudanpi Sanqi	39
Luteolin	MOL000006	Honghua, Machixian	20
Arachidonic acid	MOL001439	Machixian	15
Baicalein	MOL002714	Chishao	15
Kaempferol	MOL000422	Mudanpi, Machixian, Huangqi, Honghua	12
7-*o*-methyl isomucronulatol	MOL000378	Huangqi	8
Formononetin	MOL000392	Huangqi	8
Prangenidin	MOL003588	Baizhi, Fang feng	7
Calycosin	MOL000417	Huangqi	7
Phellopterin	MOL002644	Baizhi, Fang feng	5

TCM = traditional Chinese medicine.

### 3.4. PPI network and topology analysis

**Figure 4.**
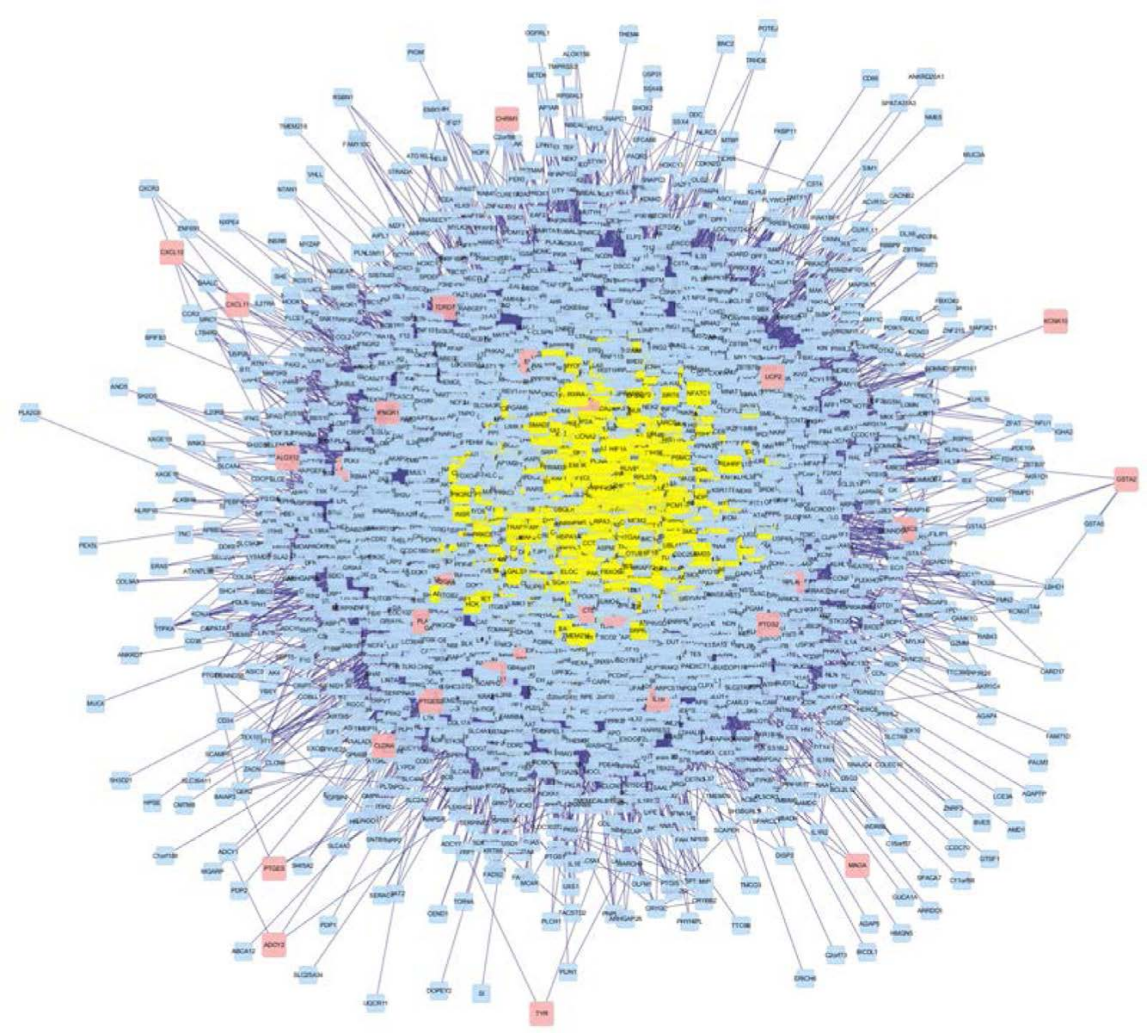
PPI network diagram. Yellow is the protein with DC >61. DC = degree centrality, PPI = protein–protein interaction.

**Figure 5.**
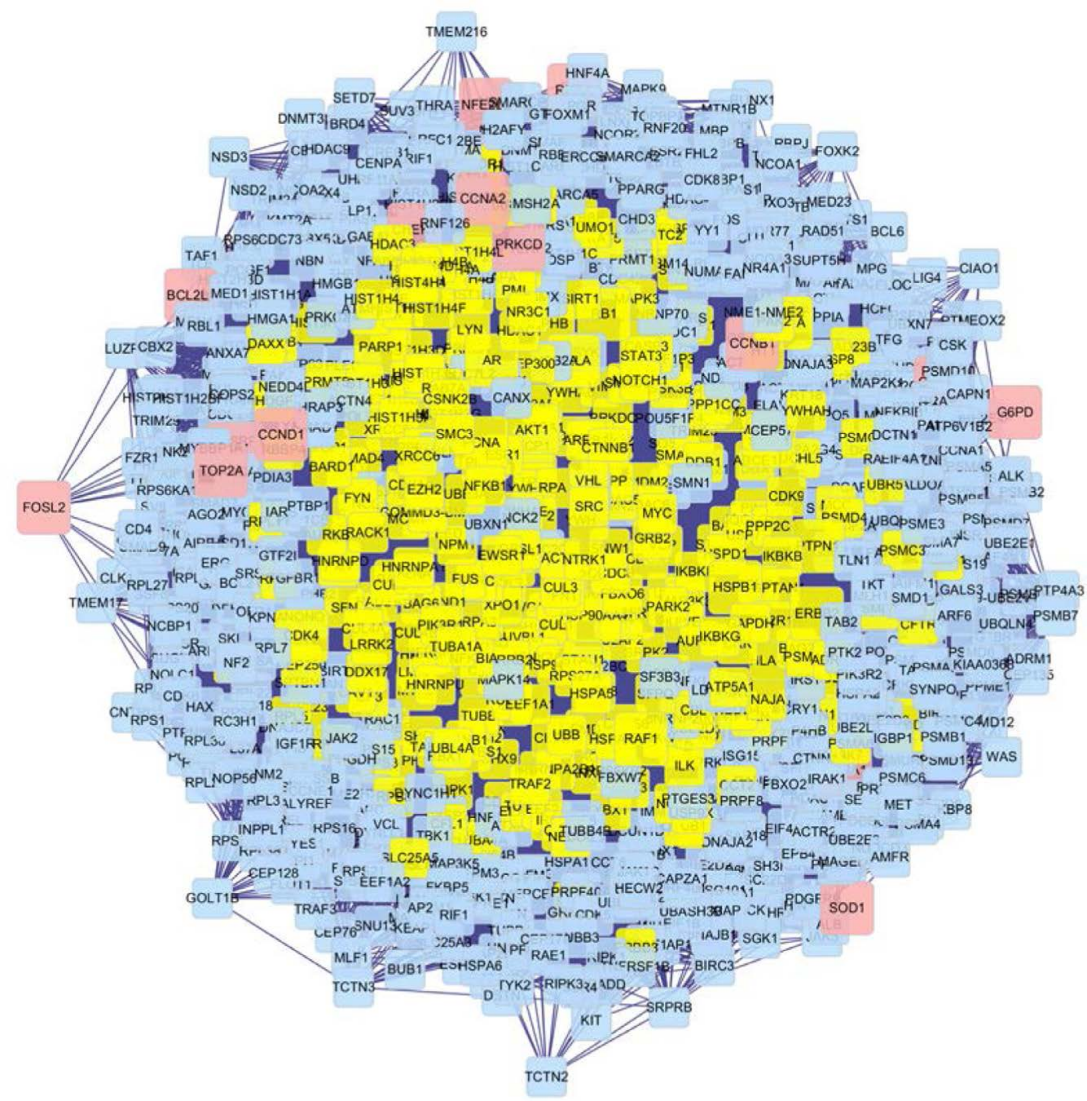
PPI subnetwork. Yellow is the protein with BC >600. BC = betweenness centrality, PPI = protein–protein interaction.

**Figure 6.**
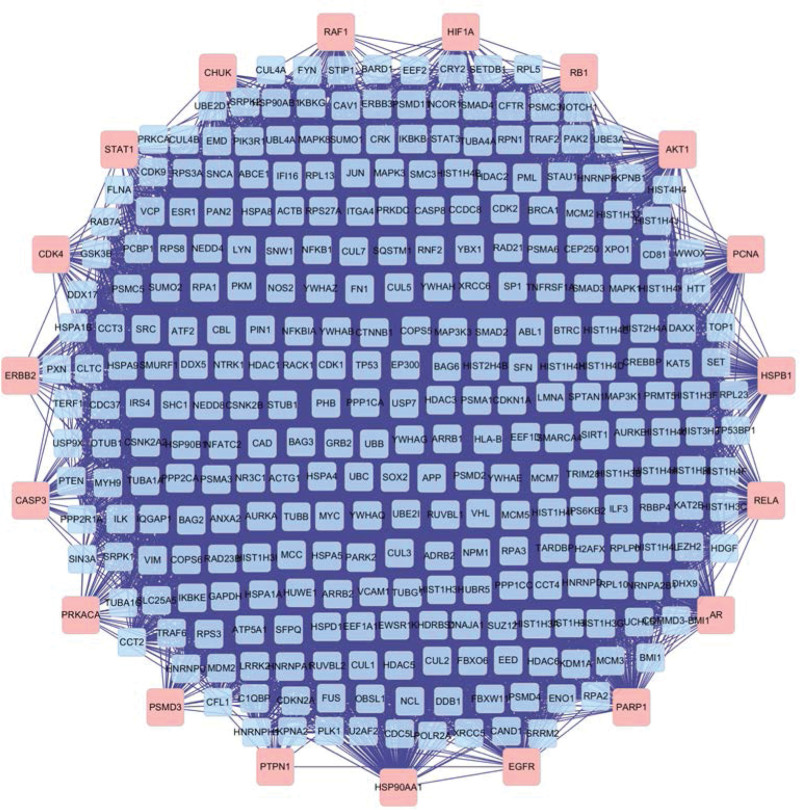
PPI network (DC >61 and BC >600). BC = betweenness centrality, DC = degree centrality, protein–protein interaction.

The obtained 4295 related proteins were drawn into PPI network with Cytoscape. A total of 947 proteins were obtained by screening with DC >61, and the qualified proteins were labeled yellow (Fig. 4), in which the maximum value of DC was 1061. After further screening with BC >600, a total of 320 key proteins were obtained. The qualified proteins were labeled yellow (Fig. 5), of which the maximum value of BC was 27,738, and finally, PPI network of key proteins was obtained (Fig. 6). According to the network topology parameters, the BC of neurotrophic receptor tyrosine kinase 1 (NTRK1), tumor protein P53 (TP53), cullin 3 (CUL3), estrogen receptor 1 (ESR1), ubiquitin C (UBC), and EGFR is 12 times greater than the median BC of the whole network (1209), which is predicted to be the core protein acted by bailing tablet.

### 3.5. GO and KEGG enrichment analysis

**Figure 7.**
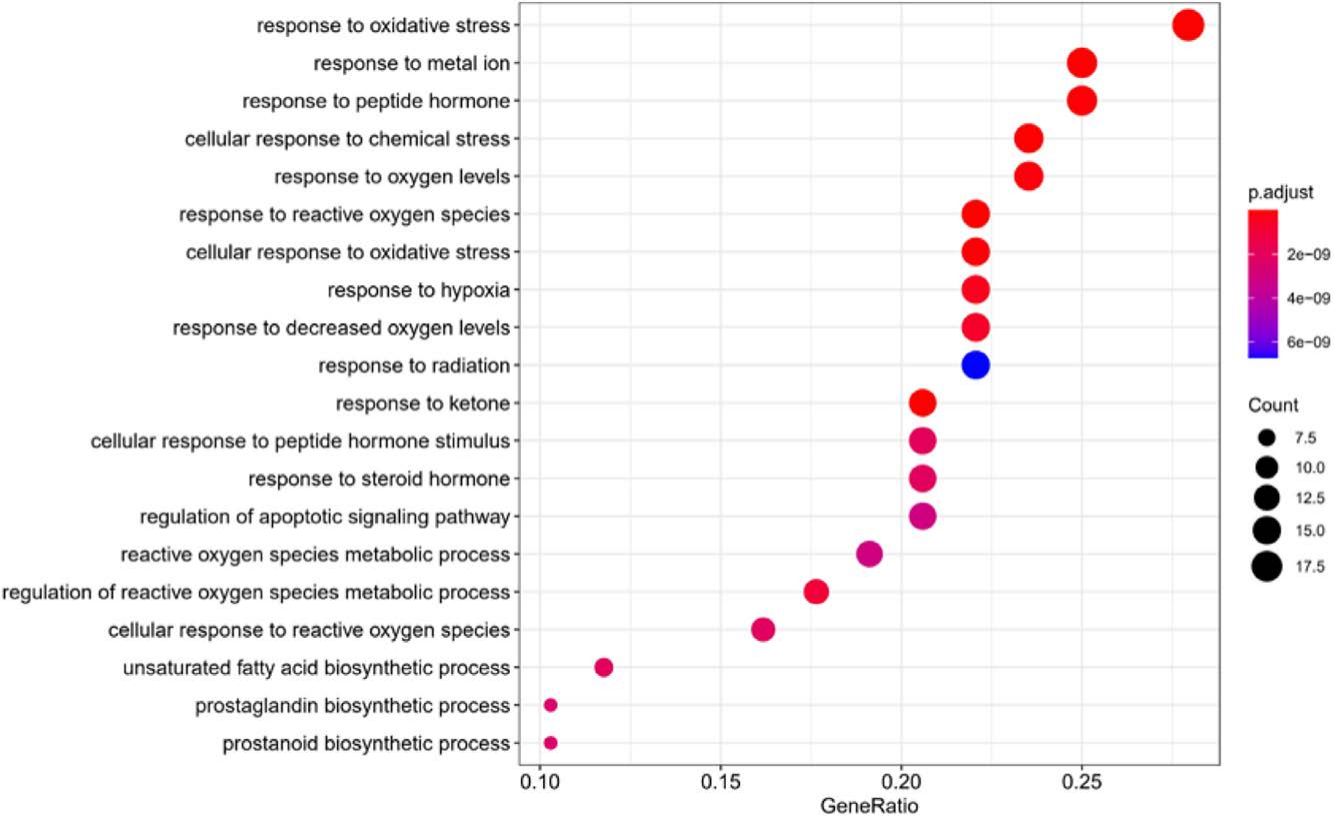
GO function enrichment analysis bubble diagram (BP). Abscissa: gene scale; ordinate: name of GO; the circle size represents the number of enriched genes; color represents the significance of enrichment. BP = biological process, GO = gene ontology.

**Figure 8.**
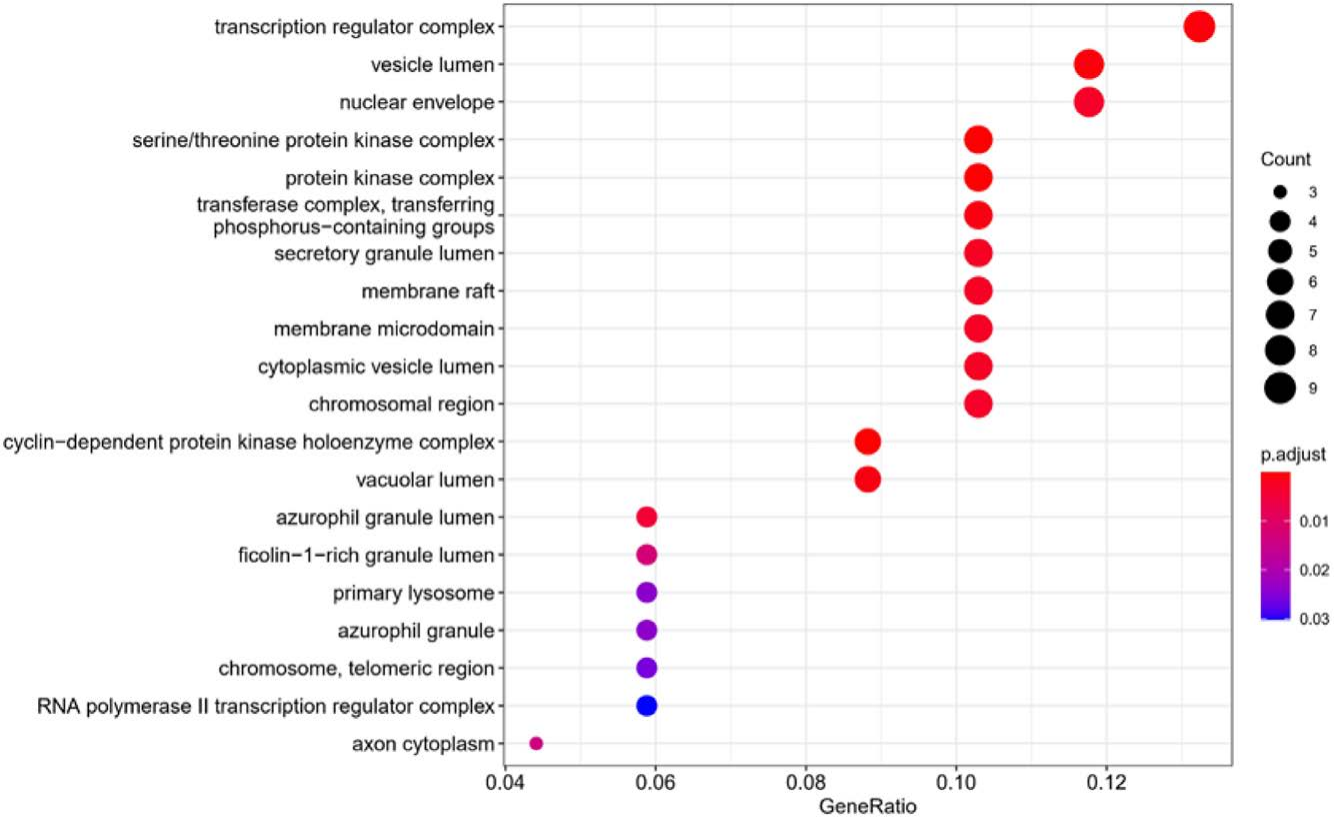
GO function enrichment analysis bubble diagram (CC). Abscissa: gene scale; ordinate: name of GO; the circle size represents the number of enriched genes; color represents the significance of enrichment. CC = cell component, GO = gene ontology.

**Figure 9.**
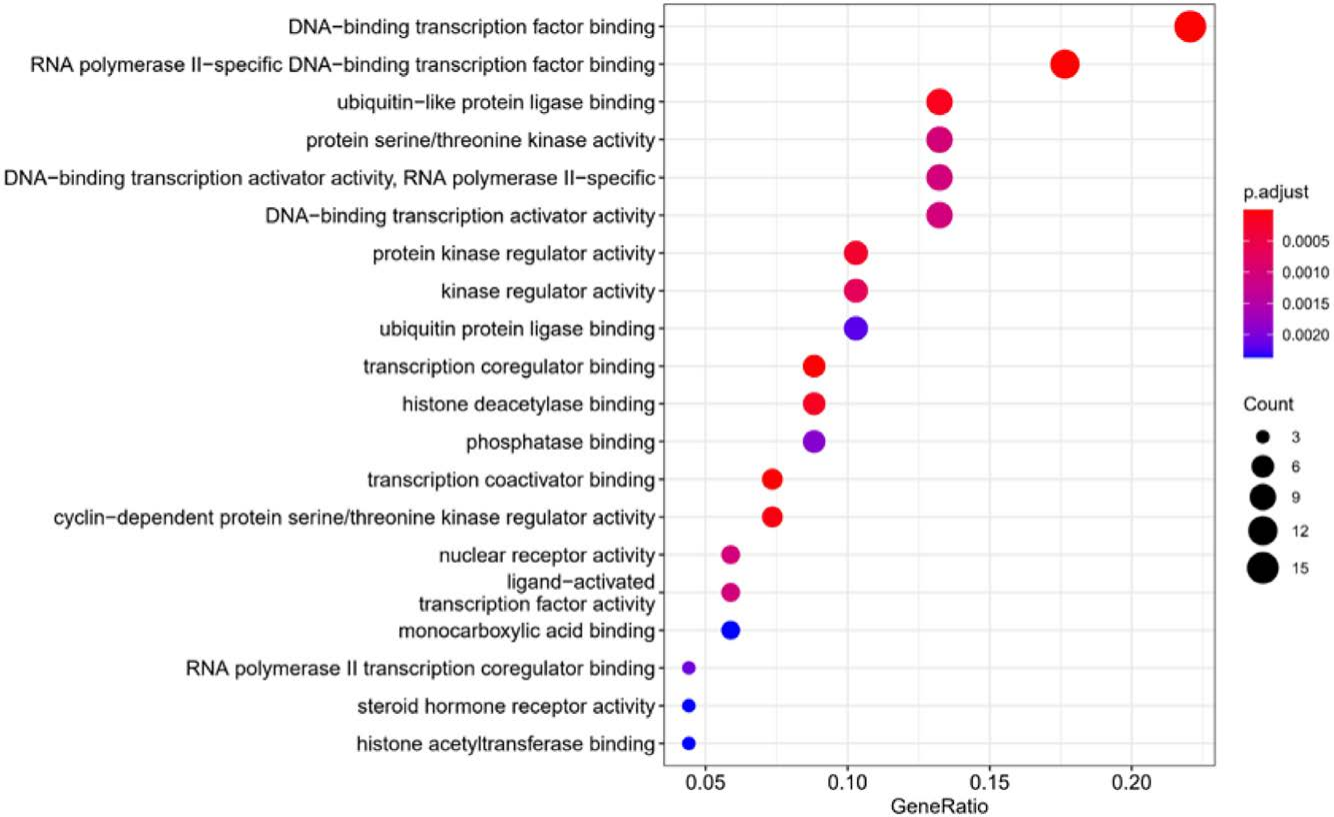
GO function enrichment analysis bubble diagram (MF). Abscissa: gene scale; Ordinate: name of GO; the circle size represents the number of enriched genes; color represents the significance of enrichment. GO = gene ontology, MF = molecular function.

Go function enrichment analysis of BP, cell component, and MF was carried out for the target, and bubble diagrams were made (Figs. 7–9). These core proteins significantly affect the BPs in response to oxidative stress, cell response to reactive oxygen species, regulation of reactive oxygen species metabolic process, cell response to chemical stress, reactive oxygen species metabolic process, and so on. Furthermore, it affects cell composition in chromosome, telomere region, RNA polymerase II transcription regulatory complex, secret granule lumen, membrane microdomain, and so on. Moreover, it affects MF in DNA-binding transcription factor binding, transcription coactivator binding, RNA polymerase II–specific DNA-binding transcription factor binding, cyclin-dependent protein serine/threonine kinase regulator activity, etc.

### 3.6. KEGG pathway enrichment analysis and KEGG network

**Figure 10.**
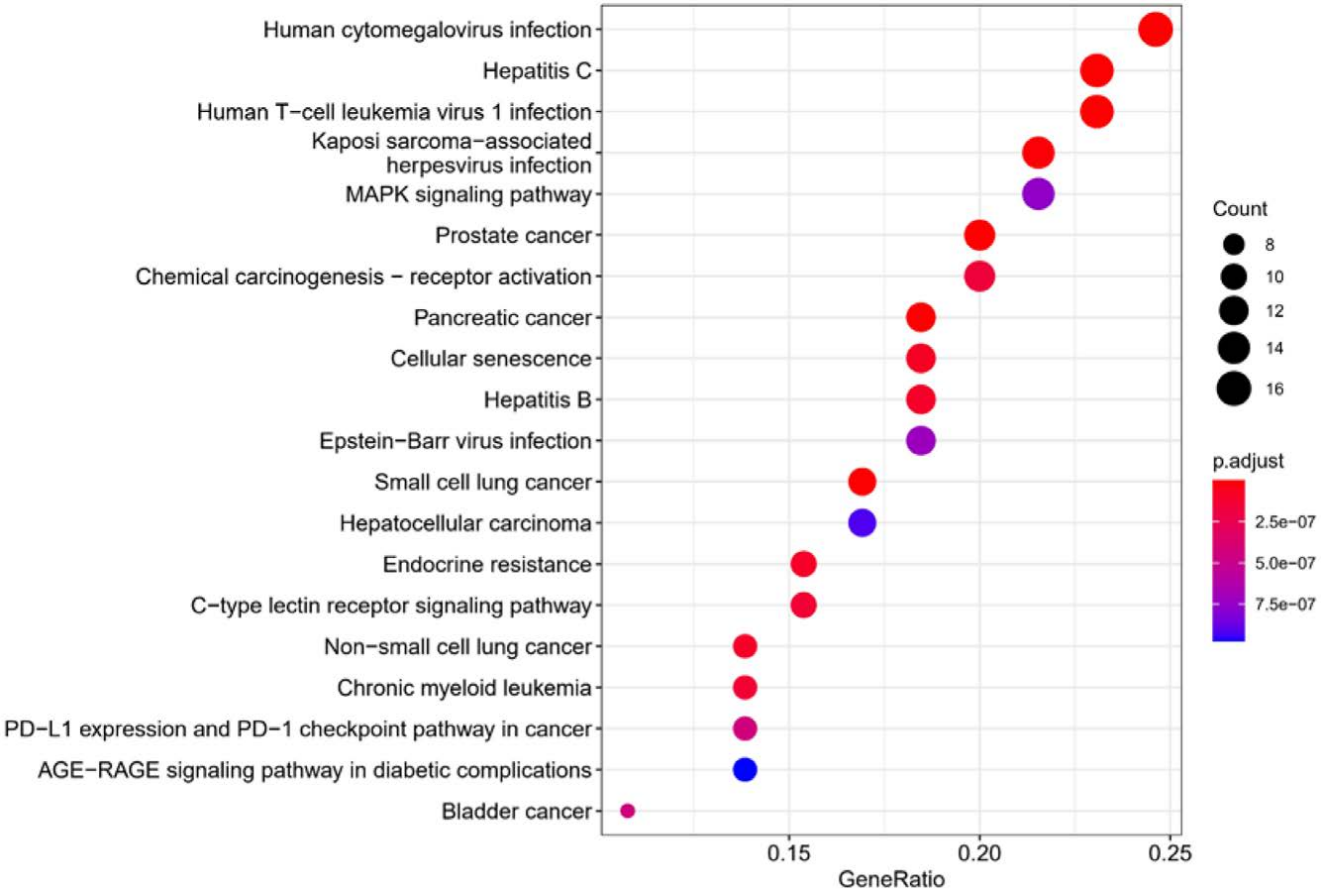
Bubble diagram of KEGG enrichment analysis. Abscissa: gene proportion, ordinate: KEGG name, circle size: number of genes in enrichment pathway, color: significance of enrichment. KEGG = Kyoto encyclopedia of genes and genomes, MAPK = mitogen-activated protein kinase.

**Figure 11.**
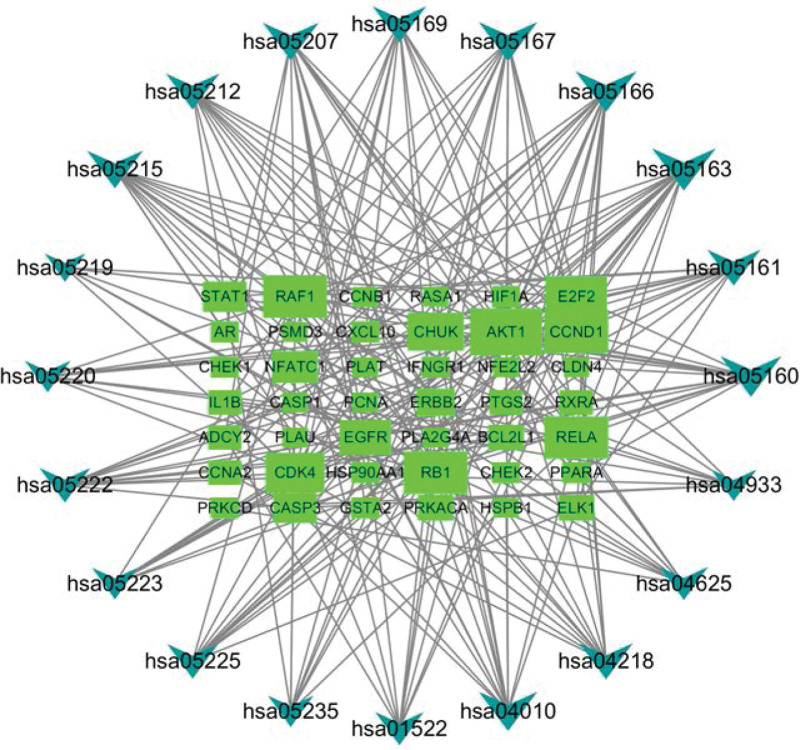
KEGG network. The periphery is the enriched KEGG pathway of the first 20, and the middle is the corresponding gene. The connecting line indicates that the gene belongs to this pathway. The more connecting lines between genes and pathways, the larger the graphic area. KEGG = Kyoto encyclopedia of genes and genomes.

KEGG pathway enrichment analysis revealed that there were 135 potential pathways for bailing tablet to treat vitiligo, and the bubble diagram was made according to the first 20 pathways (Fig. 10). KEGG network (Fig. 11) includes the first 20 pathways, and corresponds to 42 genes and 183 nodes, of which AKT1, CCND1, E2F2, RB1, RELA, CDK4, and other genes appear the most. It involves PI3K-Akt signaling pathway, chemokine signaling pathway, mitogen-activated protein kinase (MAPK) signaling pathway, TOLL receptor signaling pathway, Janus kinase signal transducer and activator of transcription (JAK-STAT) signaling pathway, T cell receptor signaling pathway, apoptosis, autophagy, nucleotide oligomerization domain receptor signaling pathway, melanin production, p53 signaling pathway, helper T cell (Th)17 cell differentiation, interleukin (IL)-17 signaling pathway, etc.

### 3.7. Molecular docking results and analysis

**Figure 12.**
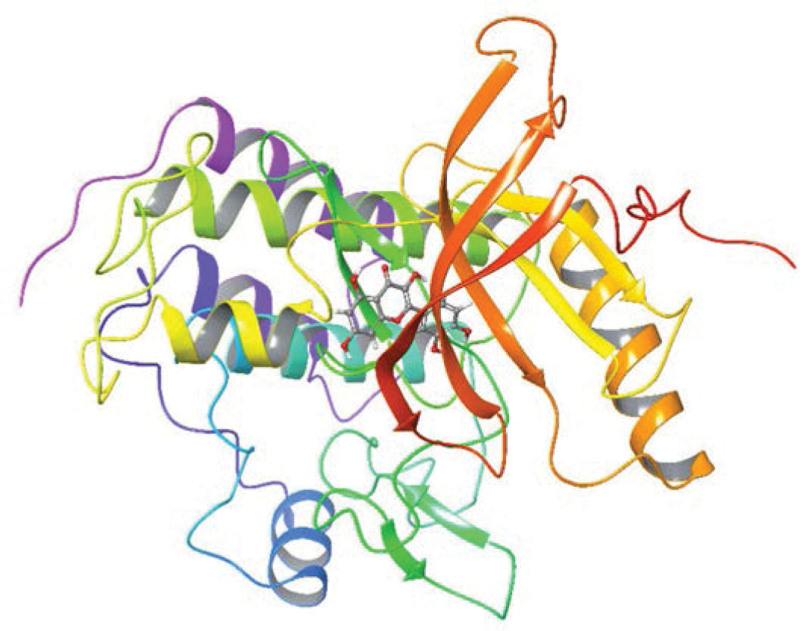
Molecular docking relationship between NTRK1 and quercetin. NTRK1 = neurotrophic receptor tyrosine kinase 1.

**Figure 13.**
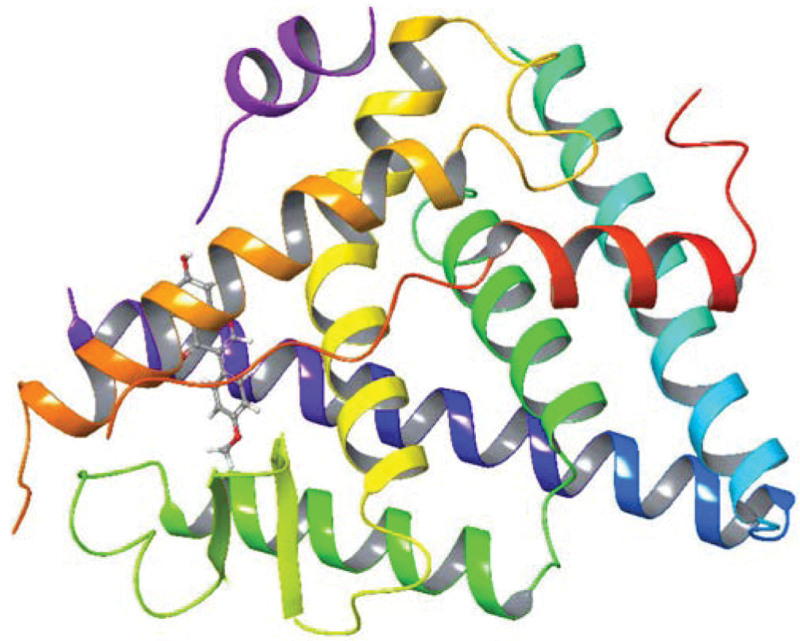
Molecular docking relationship between ESR1 and formononetin. ESR1 = estrogen receptor 1.

**Figure 14.**
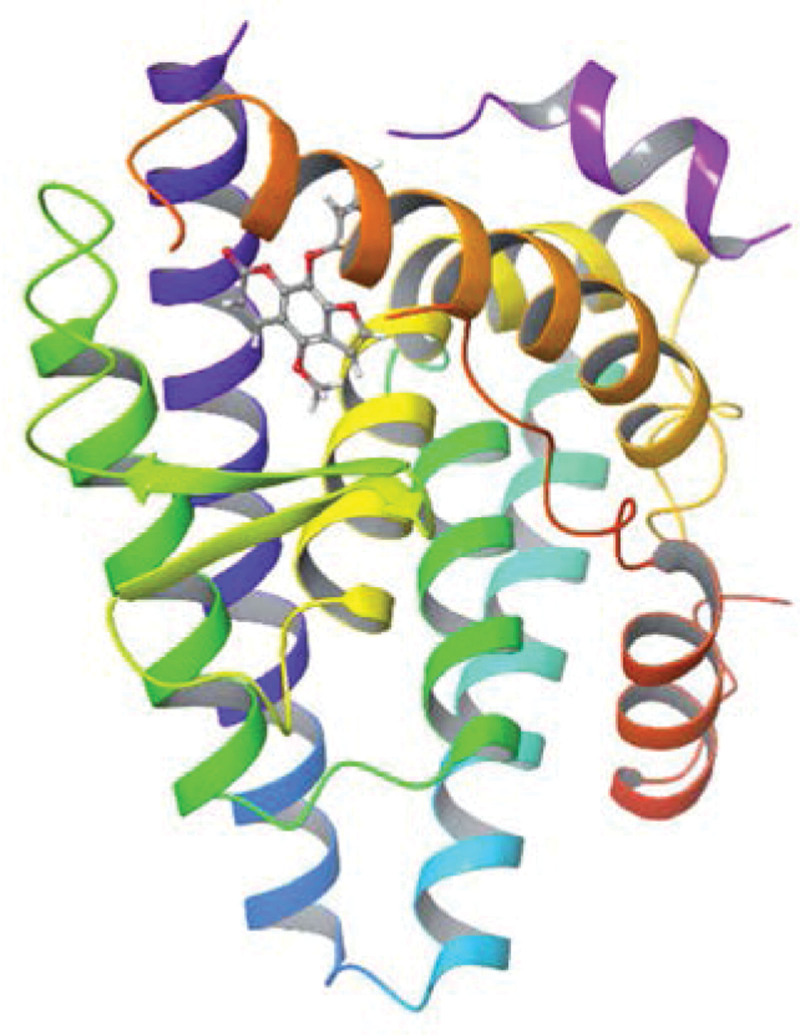
Molecular docking relationship between ESR1 and phellopterin. ESR1 = estrogen receptor 1.

In order to verify the accuracy of results in our study, 6 core proteins (NTRK1, TP53, CUL3, ESR1, UBC, and EGFR) screened by PPI network were docked by Schrodinger software, which showed good docking ability (Figs. 12–14). According to previous studies, results are meaningful when the docking score between components and proteins is <–5 kcal/mol.^[10]^ The absolute value of docking score >6 is plotted into a chart. The docking score (kcal/mol) indicates the binding force between the compound and the target protein. The smaller the binding energy is, the better the combination of the 2 is (Table 2).^[11,12]^ Among them, ESR1 protein (protein number: 7msa) has the best binding ability. It is predicted that ESR1 protein may play an important regulatory role.

**Table 2:** Molecular docking results of target proteins and active compounds.

Proteins	Test compounds	Affinity (kcal/mol)
NTRK1	Quercetin	–8.327
NTRK1	Prangenidin	–7.759
NTRK1	Formononetin	–7.336
NTRK1	Kaempferol	–6.939
NTRK1	Phellopterin	–6.789
NTRK1	Luteolin	–6.496
NTRK1	Arachidonic acid	–6.168
ESR1	Formononetin	–9.178
ESR1	Prangenidin	–8.180
ESR1	Baicalein	–8.077
ESR1	Phellopterin	–7.932
ESR1	Kaempferol	–7.771
ESR1	Luteolin	–7.701
ESR1	Calycosin	–7.463
ESR1	Quercetin	–7.238
ESR1	Arachidonic acid	–7.238
ESR1	7-*o*-methyl isomucronulatol	–7.097
UBC	Quercetin	–6.028
EGFR	Luteolin	–6.149
EGFR	Kaempferol	–6.054
EGFR	Quercetin	–6.029

EGFR = epidermal growth factor receptor, ESR1 = estrogen receptor 1, NTRK1 = neurotrophic receptor tyrosine kinase 1, UBC = ubiquitin C.

## 4. Discussion

As a common acquired, localized, or generalized skin depigmentation disease, vitiligo occurs at any age and any part, without conscious symptoms. TCM doctors believe that the main etiology and pathogenesis of vitiligo is “blood deficiency and wind dryness.” The treatment is mainly to tonify the liver and kidney, nourish blood, activate blood, and dispel wind. In this article, the mechanism of bailing tablet in the treatment of vitiligo was explored from a multitude of angles and levels by using “traditional Chinese medicine compound target” network, PPI network, GO, KEGG enrichment, molecular docking, and other technologies.

According to the “traditional Chinese medicine compound target” network, quercetin, baicalein, luteolin, kaempferol, arachidonic acid, calycosin, formononetin, prangenidin, phellopterin, and 7-*o*-methylisoxylitol are the main active components of bailing tablet, which acts synergistically on multiple targets to exert curative effects. According to the node characteristics of candidate compounds, a compound often corresponds to multiple targets, reflecting the multitarget treatment of vitiligo with bailing tablet. Furthermore, one compound can also come from a variety of herb, reflecting the advantages of compatibility of TCM. Quercetin belongs to flavonols. It was found that H_2_O_2_ could induce the expansion of endoplasmic reticulum and hinder the output of functional tyrosinase from the endoplasmic reticulum of melanocytes, thus resulting in the pathogenesis of vitiligo. Quercetin can reduce a series of oxidation reaction processes mediated by H_2_O_2_, and finally reduce the incidence of vitiligo.^[13]^ Baicalein is a flavonoid active substance extracted from *Scutellaria baicalensis*, and it can upregulate the expression of GPX4 gene, reduce the level of TFR1, inhibit the iron death of melanocytes, and enhance the antioxidant defense ability of human vitiligo melanocytes.^[14]^ Luteolin is a natural flavonoid with many pharmacological activities, such as anti-inflammatory, antiallergic, antioxidant, and so on. Inflammation and oxidation should play a major role in the pathogenesis of vitiligo. IL-8 is a major chemokine in inflammatory skin diseases. At the same time, the data proved that the expression of IL-8 gene in the skin of patients with vitiligo increases, thereby resulting in pathogenesis. With strong anti-inflammatory and antioxidant effects, luteolin can prevent the expression and release of IL-8 gene in melanocytes, which can reduce the incidence rate of vitiligo.^[15]^ Kaempferol is a flavonoid compound with anti-inflammatory and antioxidant effects, and it has been proved to be a melanopoietin.^[16]^ A variety of compounds contained in 11 herbs of bailing tablet exert their efficacy from different levels, such as cell, factor, protein synthesis, etc. These results are consistent with the prediction results of this study and could prove the reliability of the results.

According to the PPI network of bailing tablet, the degree value of TP53, ESR1, and other proteins is the largest, which may be an important target for its curative effect. The overexpression of TP53 (cell tumor antigen p53) can protect patients with vitiligo from photochemical damage and change the migration ability of melanocytes, so as to improve pigmentation in patients with vitiligo.^[17]^ Oxidative stress is the main pathogenesis of vitiligo. Although ESR1 could not increase the number of melanocytes, it could protect melanocytes from oxidative stress-induced cytotoxicity, thus cutting off the pathogenesis path of vitiligo.^[18,19]^ Our results are similar to previous studies. KEGG enrichment analysis showed that bailing tablet mainly regulates immune inflammation, endocrine function, cell generation, apoptosis, autophagy, and other related pathways in the treatment of vitiligo. Among them, bailing tablet regulates the main pathways of melanogenesis, including p38/MAPK, CAMP/PKA, and PI3K/Akt. P38/MAPK can increase the expression of microphthalmia-associated transcription factor (MITF) and tyrosinase, thereby inducing melanogenesis.^[20]^ Increased intracellular levels of CAMP can activate protein kinase (PKA), and CAMP can activate protein kinase (PKA) to phosphorylate CAMP-response element-binding protein and CAMP-response element binding protein-binding protein, thereby increasing the expression of MITF.^[21]^ PI3K/Akt signaling pathway inhibits melanogenesis by reducing the expression of tyrosinase, MITF and transient receptor potentical.^[22]^ In addition, bailing tablet also play a potential role in immune inflammation by mediating the differentiation of Th1, Th2, and Th17 and regulating the secretion of inflammatory factors such as IL-17, IL-6, tumor necrosis factor-α, and IL-1, which can reduce melanocyte injury.^[23]^ The expression of Th1 chemokine CXCL10 is significantly increased in patients with vitiligo, which is expected to become a new target for the treatment of vitiligo.^[24]^ In addition, JAK is also the main pathway of bailing tablet. There are literature studies on interferon (IFN)-γ after treatment, and it can lead to a concentration-dependent increase in chromatin condensation and nuclear fragmentation, which indicates that IFN- γ can directly induce mononuclear cell apoptosis.^[24]^ JAK-STAT is IFN- γ signal path, and some cytokines, such as leukocytes and IFNs, use the JAK-STAT pathway to transmit the signal from the cell membrane to the nucleus. Then the JAK protein was turned into an active protein, the phosphate STAT protein was darkened, and finally the nucleus was used to regulate the expression of related genes.^[2]^ Therefore, JAK inhibitors are often used to treat vitiligo. For example, patients with generalized vitiligo were treated with tofacitinib.

According to the results of protein topology analysis and previous research support, we selected core proteins, including NTRK1, TP53, CUL3, ESR1, UBC, and epidermal growth factor receptor (EGFR), for molecular docking. The results revealed that the main compounds could bind well with core proteins. The docking score of ESR1 and NTRK1 is large, which further verifies that they may be the main protein targets of bailing tablet.

Our study has some limitations. On the one hand, network pharmacology is a bioinformatics analysis based on databases. Due to the limitations of the databases, there may be deviations in the prediction target, and improving the database construction will enhance the credibility of the results. On the other hand, we analyzed the potential mechanisms of bailing tablet in the treatment of vitiligo, and the results need to be further verified by cell/animal experiments or clinical experiments.

## 5. Conclusion

Bailing tablet plays a role in anti-inflammatory, antioxidation, and inhibition of melanocyte death. A compound can act on multiple targets related to the treatment of vitiligo, and each target can be related to multiple pathways. Clinically, bailing tablet is effective in the treatment of vitiligo, but the mechanism of action is not clear. This article explored the mechanism of bailing tablet in the treatment of vitiligo through multicomponent, multitarget, and multichannel through TCM network pharmacology and molecular docking technology. It highlights the scientificity and effectiveness of bailing tablet, provides guiding significance for clinicians in the treatment of vitiligo, and also provides new ideas for further exploring the mechanism of bailing tablet.

## Author contributions

Conceptualization and data curation: Jinming Li,Meng Yang.

Funding acquisition and investigation: Yeqiang Song.

Methodology and project administration: Jinming Li.
